# Contrasting impacts of two weed species on lowbush blueberry fertilizer nitrogen uptake in a commercial field

**DOI:** 10.1371/journal.pone.0215253

**Published:** 2019-04-12

**Authors:** Charles Marty, Josée-Anne Lévesque, Robert L. Bradley, Jean Lafond, Maxime C. Paré

**Affiliations:** 1 Laboratoire d’écologie végétale et animale, Département des sciences fondamentales, Université du Québec à Chicoutimi, Saguenay, QC, Canada; 2 Département de biologie, Université de Sherbrooke, Sherbrooke, QC, Canada; 3 Agriculture and Agri-Food Canada, Soils and Crops Research and Development Centre, Normandin, QC, Canada; Consiglio per la Ricerca e la Sperimentazione in Agricoltura, ITALY

## Abstract

Numerous studies have speculated that lowbush blueberry (*Vaccinium angustifolium****)*** is less efficient than weed species at taking up inorganic nitrogen (N) derived from fertilizers, thus raising questions as to the effectiveness of N fertilization in commercial fields. However, competition for acquiring N as well as specific interactions between blueberry and companion weeds characterized by contrasted functional traits remain poorly documented. Here, we assessed fertilizer-derived N acquisition efficiency and biomass production in lowbush blueberry and two common weed species that have different functional traits—sweet fern (*Comptonia peregrina*), a N_2_-fixing shrub, and poverty oat grass (*Danthonia spicata*), a perennial grass—in a commercial blueberry field in Québec, Canada. In 2015, ^15^N-labelled ammonium sulfate was applied at a rate of 45 kg ha^-1^ to 1 m^2^ field plots containing lowbush blueberry and one of the two weeds present at several different density levels (0 to 25 plants m^-2^). In 2016, each plot was harvested to determine vegetative biomass and the percentage of fertilizer-derived N recovered (PFNR) in each species. The PFNR was higher in blueberry (24.4 ± 9.3%) than in sweet fern (13.4 ± 2.6%) and poverty oat grass (3.3 ± 2.9%). However, lowbush blueberry required about four times more root biomass than sweet fern and poverty oat grass to uptake an equivalent amount of N from ammonium sulfate. The PFNR in poverty oat grass increased with plant density (from 0.8% to 6.4% at 2–3 and >6 plants m^-2^, respectively), which resulted in a decrease in blueberry’s PFNR (from 26.0 ± 1.4% to 8.6 ± 1.8%) and aboveground vegetative biomass production (from 152 ± 58 to 80 ± 28 g m^-2^). The increase in biomass production and N content in sweet fern with increasing plant density was not accompanied by an increase in PFNR (29.7 ± 8.4%), suggesting an increasing contribution of atmospherically-derived N. This mechanism (i.e., N sparing) likely explained blueberry’s higher biomass production and N concentration in association with sweet fern than with poverty oat grass. Overall, our study confirms lowbush blueberry low efficiency (on a mass basis) at taking up N derived from the fertilizer as compared to weeds and reveals contrasted and complex interactions between blueberry and both weed species. Our results also suggest that the use of herbicides may not be necessary when poverty oat grass is present at a low density (<15 plants of poverty oat grass m^-2^) and that adding inorganic N fertilizer is counterproductive when this species is present at a high density as it takes up as much fertilizer as lowbush blueberry.

## Introduction

Lowbush blueberry (*Vaccinium angustifolium****)*** is an ericaceous shrub native to eastern North America that grows on disturbed sites [1,2]. It is an economically significant crop in Québec, Canada’s Maritimes Provinces and Maine [3], where it is managed for its fruits from naturally occurring plant populations [4]. Commercial lowbush blueberry fields are often developed on forest clear cuts where plants regrow from their large rhizomatic network and clones can spread naturally [2,5,6]. Lowbush blueberry fruits are produced generally through a 2-year crop cycle; the shrubs are pruned to near ground level in the first year (vegetative year) to stimulate new shoot production, and the plants bloom and produce fruits in the second year [6,7]. Lowbush blueberry lives in symbiosis with ericoid mycorrhizal fungi, which gives the plant access to otherwise inaccessible nutrients, especially from recalcitrant soil organic matter [8–10]. Although lowbush blueberry is well adapted to low nutrient availability and has low N requirements, repeated use of inorganic or organic fertilizers has been shown to increase leaf nutrient concentrations and growth [7,11–14] as well as fruit yields [11,14–16]. Weed control is, however, often necessary for fertilizers to be efficient [4,6,11,13,17,18].

The weed flora in lowbush blueberry stands consists of a broad range of native herbaceous and woody perennial species, the specific taxa varying with soil type, moisture and fire history [6]. The Polygonaceae sheep sorrel (*Rumex acetosella* L.), the Myricaceae sweet fern (*Comptonia perigrina* L.), the Poaceae poverty oat grass (*Danthonia spicata* L. P. Beauv. Ex Roem. & Schult.) and the Ericaceous species sheep laurel (*Kalmia angustifolia* L.), Canada rhododendron (*Rhododendron canadense* L.) and Labrador tea (*Ledum groenlandicum* Oeder) are among the most common weed species found in commercial blueberry fields of Québec and other Canadian provinces [6,19–21]. Although several studies have shown the detrimental effect of weeds on blueberry’s fertilizer uptake and yield [6,11,22], the specific interactions between lowbush blueberry, weed species and weed density have not been investigated in detail. However, it is likely that the specific functional traits and morphology of weed taxa control these interactions and, therefore, lowbush blueberry’s growth and fruit production. The presence of N_2_-fixing species often increases N availability for companion species both in cultivated [23–27] and natural ecosystems [28–30] due to direct or indirect N transfers from the N-fixing to the companion species [29] or due to “N sparing” [28], i.e. lower competition of N-fixing species for soil inorganic N by their ability to acquire N from the atmosphere. Therefore, it is likely that the interaction of blueberry with sweet fern, an actinorhizal N-fixing shrub (0.3–1.5 m in height) with a deep root system [31–33] is different than its interaction with poverty oat grass, a grass having a dense and superficial root system [34]. Sweet fern, like several Myricacea species, develops cluster roots, allowing this species to colonize sterile soils, such as abandoned fields and pine barrens [33]. These traits, combined with its ability to acquire N from the atmosphere through its symbiotic association with the actinobacterium *Frankia*, increase its fitness in habitats marked by low nutrient availability and a short growing period, such those found in the boreal zone, and make it one of the most serious weed problems in Canada’s commercial lowbush blueberry fields [33]. The development of cluster roots by this species also provides the plant the ability to access scarce and non-labile essential nutrients such as inorganic P [33,35]. Therefore, while sweet fern may not be a strong competitor for N fertilizer, it may impact lowbush blueberry growth by competing for other resources such as P, that can be a limiting factor for blueberry’s growth and yields [36]. In contrast, N fertilization in stands marked by a high density of poverty oat grass—a species native to North America [37] and often dominant grass in pastures of southern Québec and the Maritime provinces [34]—may be counterproductive. This species has a dense, fibrous and superficial root system that may allow it to capture most N applied to the soil surface. Fertilization may thus increase weed growth rather than favouring a greater lowbush blueberry yield. Such a detrimental effect of fertilization has been observed in southern Estonia lowbush blueberry fields, where liming increased soil pH and resulted in increased weed density and decreased blueberry growth [38]. Due to the economic significance of lowbush blueberry in northeastern North America and the potential growth of this market in several countries, it is important to better understand the N requirements of lowbush blueberry and how this plant interacts with neighbouring species, so as to adapt fertilization and cultural practices.

In this study, we investigated how lowbush blueberry grows and competes for acquiring N fertilizer in mixtures with sweet fern or poverty oat grass in a commercial blueberry field in the Lac-Saint-Jean region, Québec, Canada. Experimental plots were delimited within a commercial lowbush blueberry stand as a function of the presence/absence and density of sweet fern and poverty oat grass. In particular, we assessed i) the proportion of the fertilizer recovered in lowbush blueberry versus weed species, ii) the impact of weed density on blueberry’s acquisition of fertilizer N and biomass production, and iii) the weed species having the most detrimental effect on blueberry’s acquisition of N and biomass production. We hypothesized that lowbush blueberry would be a poor competitor for fertilizer compared to the two studied weed species and that increasing weed density would decrease lowbush blueberry fertilizer-derived N uptake and biomass production as a result of competition for N and other resources. We also hypothesized that poverty oat grass would compete more strongly with lowbush blueberry for acquiring N from the fertilizer than sweet fern due to the superficial root system of poverty oat grass and sweet fern’s ability to acquire N from the atmosphere.

## Material and methods

### Study site

We conducted this experiment in a commercial lowbush blueberry field in Saint-Eugène d’Argentenay in the Saguenay–Lac-Saint-Jean (SLSJ) region, Québec, Canada (48°59’N, 72°18’W; 163m a.s.l.). The Saint-Eugène d’Argentenay’s Coop (i.e., producers' association) granted us full permission to conduct this research work on their land. The SLSJ region contains 82% of Québec’s commercial lowbush blueberry surface area [3]. Soils in the lowbush blueberry stands are podzols, characterized by fine sand originating from fluvioglacial deposits and low water retention capacity; they are thus non-fertile [39]. Soil pH in the study field is moderately acidic (pH of ~5) but characterized by high Al concentrations and low CEC (~17 meq/100g) and base saturation (~5%). The organic matter content in the mineral horizons was ~4% (See S1 Table for details). The region is characterized by a cold and humid climate with a mean annual air temperature of 0.8°C. Mean precipitation and air temperature during the growing season (May to August) average 989 mm and 14°C, respectively. The lowbush blueberry stand was established in 2005 and is currently run over a 2-year cycle. The lowbush blueberry plants are pruned in the fall or in early spring, which results in the vegetative growth and in the development of flower buds the following growing season. This vegetative year is followed by the fruit production year. After fruit harvesting, the stand is mown again either at the end of fall or early in the following spring to start a new production cycle.

### Experimental design

The commercial field was scrutinized during the autumn of 2014 to identify areas colonized either by sweet fern or poverty oat grass. Initial weed density (number of plants m^-2^) was estimated visually, and 1 × 1 m plots were established in areas defined by four levels of plant density (D1–D4) for each weed (Table 1). A narrow trench was dug (~40 cm deep) around each experimental plot and filled with two layers of polyethylene tarpaulin to isolate root systems and to retain the maximum amount of ^15^N within the plots. The vegetation was then mowed ~0.5 cm above the soil level, according to standard management. Each of the four weed density plots were replicated three times (three blocks), resulting in a total number of 24 plots (2 weed species × 4 density levels × 3 replicates = 24 plots).

**Table 1:** Sweet fern and poverty oat grass density (plants m^-2^) of the four levels of weed density (D1-D4).

Weed density level	Sweet fern (plants m^-2^)	Poverty oat grass (plants m^-2^)
D1	0–1	0–1
D2	2–3	5–10
D3	4–5	15–20
D4	6+	25+

In early spring of 2015, the equivalent of 45 kg N ha^-1^ (i.e., the recommended N fertilization rate in the region) was added to each plot by pulverizing 2 L of a solution containing 22.5 g of dissolved ^15^N-enriched ammonium sulfate salt [(NH_4_)_2_SO_4_; 21% N; 5% ^15^N]. No pesticide was used during the study period. Sweet fern and poverty oat grass were the only weed species present in the experimental plots.

### Sampling and analyses

In August 2016, the vegetative biomass of the two weed species (sweet fern and poverty oat grass) and lowbush blueberry was harvested from each plot (~0.5 cm above the soil level) and taken to the lab. Belowground biomass was collected from within a 50 × 50 cm frame that was placed at the center of each plot. A fine trench was dug around the frame to a depth of ~15–20 cm. A wood board was thrust with a hammer ~15–20 cm beneath the surface to collect the entire rhizosphere within the 50 x 50 cm plot. The 50 × 50 × 15–20 cm samples were then wrapped with plastic sheets and taken to the lab. Belowground biomass was sorted among species and rinsed with demineralized water. The aboveground and belowground biomass of each plot was then dried at 60°C for 72 h, weighed and then ground using a cutting mill (Pulverisette 19, Fritsch, Idar-Oberstein, Germany). One subsample (~10 g DW) per plot was randomly taken from this roughly ground plant biomass sample and ground to a finer powder with a ball mill (Mixer Mill MM 200, Retsch, Haan, Germany). About 6 mg of this fine powder was then wrapped in tin capsules and sent to the Centre de recherche en géochimie et géodynamique (GEOTOP; UQÀM, Québec, Montréal) for total N and ^15^N analyses. Total N was measured with an automatic elemental analyzer in continuous flow mode (Vario Micro Cube, Elementar, Germany). N isotopic ratios were measured using an elemental analyzer in continuous flow mode, coupled to an isotope ratio mass spectrometer (IRMS; Micromass Isoprime Isoprime 100, Cheadle, UK). Values of δ^15^N are expressed in ‰ versus air (± 0.2‰ at 1σ). Raw values were corrected with a calibration line obtained from two reference materials: urea and dogfish tissue (δ^15^N = -0.22‰ and +14.36‰, respectively). Internal reference materials were normalized to IAEA-N1, N-2, and N-3 scales for δ^15^N. A third internal reference material was used to verify the precision of the calibration (leucine δ^15^N = -0.06‰). For each species, the amount of ^15^N (A_BM_; g) in excess in plant biomass was calculated as follows:

$$A_{BM}=N\times m\times e$$ Eq 1

where *N* and *m* are plant N concentration (g N g^-1^) and dry mass (g), respectively, and *e* the ^15^N atom percent excess in plant biomass relative to the standard (atmosphere).

The percentage of fertilizer-derived N recovered (PFNR) was then calculated for each species as follows:

$$PFNR=\frac{A_{BM}}{A_{Fertilizer}}\times 100$$ Eq 2

where A_Fertilizer_ is the amount of ^15^N applied as (^15^NH_4_)_2_SO_4_ to each plot. The acquisition of ^15^N by plants was also normalized as a function of root biomass (*R*_*BM*_; μg ^15^N g^-1^ root) to estimate fertilizer uptake efficiency (*FU*_*eff*_):

$${FU}_{eff}=\frac{A_{BM}}{R_{BM}}$$ Eq 3

### Statistical analyses

Mixed model analyses were performed on i) lowbush blueberry’s PFNR and biomass with weed species (two levels) and weed density (four levels) as fixed effects and blocks (three blocks) as the random effect; and ii) *FU*_*eff*_ with species as fixed effects and blocks as the random effect. Analyses were performed in R (R Development Core Team, 2013) with the *lmerTest* package, which uses the Satterthwaite's degrees of freedom method [40]. The package *emmeans*, which is an updated version of the *lsmeans* package [41] was used to estimate marginal means and to conduct a post-hoc pairwise comparisons of least-square means among factors’ levels (Tukey’s method). Linear regression and analyses of covariance (ANCOVA) were performed between both aboveground vegetative biomass production and belowground biomass, and PFNR across species.

## Results

### Blueberry aboveground vegetative biomass production and belowground biomass

**Fig 1:**
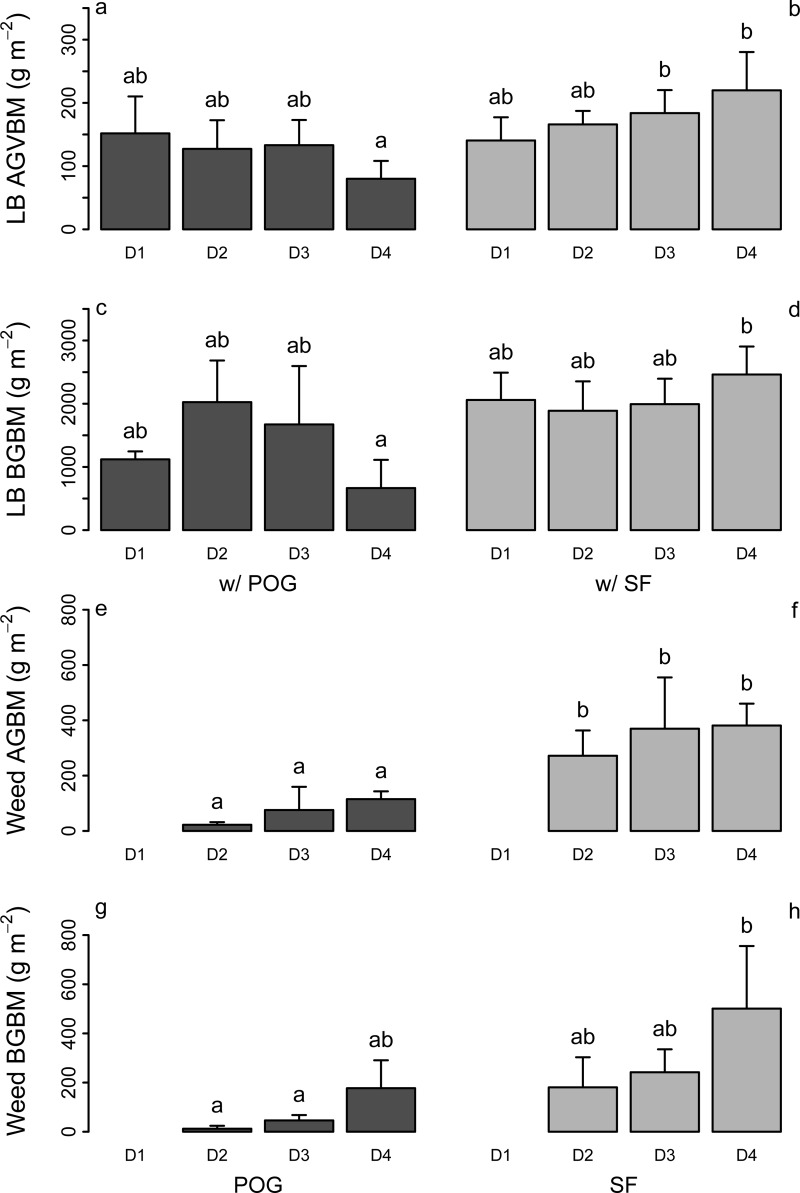
Aboveground vegetative biomass (AGVBM) production and belowground biomass (BGBM) (g m^-2^) in the three study plant species. (a–d) AGBVM and BGBM in lowbush blueberry (LB) in mixtures with poverty oat grass (POG; dark grey bars) or sweet fern (SF; light grey bars). (e–h) AGBVM and BGBM in POG (dark grey bars) or SF(light grey bars) in mixtures with LB. Values are mean ± SD (*n* = 3) and are shown for the four levels of weed density (D1-D4). Values not sharing the same letters are significantly different at *P* < 0.05.

There was a significant effect of weed species and an interaction between weed density and weed species on lowbush blueberry aboveground vegetative biomass (i.e., stems + leaves) production (Table 2). On average, lowbush blueberry aboveground vegetative biomass was 30% lower in plots with poverty oat grass (123 ± 30 g m^-2^) than in plots with sweet fern (178 ± 33 g m^-2^). In association with poverty oat grass (Fig 1A), lowbush blueberry aboveground vegetative biomass production tended to be lower at higher weed densities rather than at lower densities, whereas it tended to increase with sweet fern density (Fig 1B).

**Table 2:** Results of the mixed model analysis conducted on lowbush blueberry (LB) and weed aboveground biomass (AGBM) production (g m^-2^) and belowground biomass (BGBM; g m^-2^).

Response variable	Explanatory variable	Df	Sum of square	*F*-value	P-value
LB AGBM	Weed species	1	17873	15.02	**0.002**
	D level	3	590	0.16	0.92
	D level ×Weed species	3	17788	4.98	**0.015**
LB BGBM	Weed species	1	200020	11.94	**0.004**
	D level	3	40964	0.81	0.51
	D level × Weed species	3	196608	3.91	**0.032**
Weed AGBM	Weed species	1	223178	23.5	**4.0 10**^**−4**^
	D level	3	66635	2.3	0.12
	D level × Weed species	3	9735	0.3	0.79
Weed BGBM	Weed species	1	14792	13.9	**2.9 10**^**−3**^
	D level	3	12419	3.9	**3.7 10**^**−2**^
	D level × Weed species	3	1296	0.6	0.56

Weed species and weed density level were used as fixed effects and blocks as random effect. The table shows the results of type III analyses of variance computed via Satterthwaite's degree of freedom method. Significant effects (P<0.05) are shown in bold characters.

There was also a significant effect of weed species and an interaction between weed density and weed species on lowbush blueberry belowground biomass (Table 2). Blueberry belowground biomass was 35% lower in plots having poverty oat grass (1371 ± 600 g m^-2^) than in those having sweet fern (2101 ± 251 g m^-2^), especially in D4 plots (Fig 1C and 1D).

The aboveground vegetative biomass/belowground biomass ratio did not vary significantly among density levels and weed species. It averaged 0.11 ± 0.06 g g^-1^ in poverty oat grass and 0.09 ± 0.02 g g^-1^ in sweet fern.

### Weed above and belowground biomass

Aboveground biomass production of sweet fern was about five times higher (321 ± 132 g m^-2^) than that of poverty oat grass (64 ± 61 g m^-2^). Although the biomass tended to increase with weed density (Fig 1E to 1F), the effect of the weed density level was not significant (Table 2).

Belowground biomass was also much higher in sweet fern than in poverty oat grass (306 ± 198 g m^-2^ vs. 79 ± 95 g m^-2^; Fig 1G and 1H), but belowground biomass varied strongly among density levels (Table 2). In both species, belowground biomass increased with weed density (Fig 1G and 1H).

The aboveground biomass production/belowground biomass ratio was on average 30% higher in poverty oat grass than in sweet fern (2.0 ± 2.4 vs. 1.5 ± 1.2, respectively); however, it strongly decreased with increasing density for both species: from 3.8 ± 3.7 in D2 plots to 1.3 ± 0.9 in D3 plots to 0.81 ± 0.5 in D4 plots for poverty oat grass and from 2.4 ± 1.9 in D2 plots to 1.7 ± 0.8 in D3 plots to 0.8 ± 0.2 in D4 plots for sweet fern.

### N concentrations in aboveground and belowground biomass

**Fig 2:**
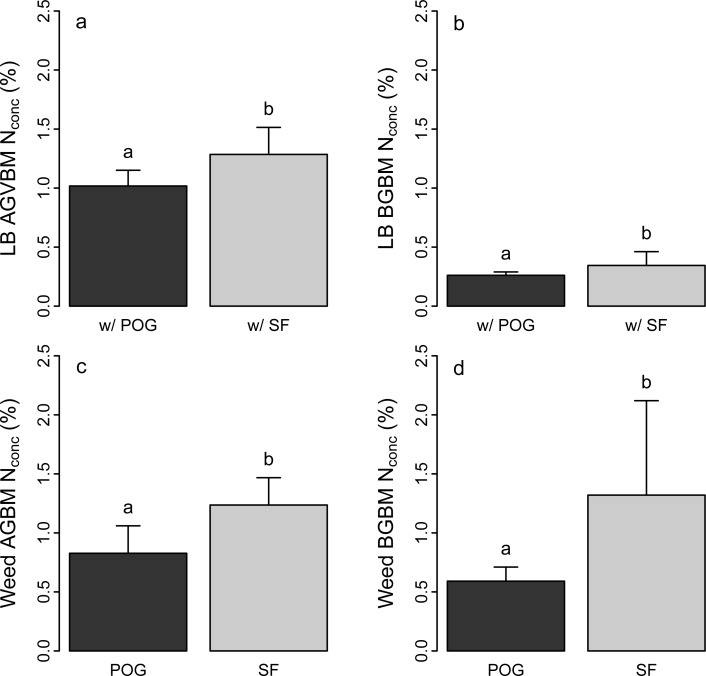
Total N concentration (%) in aboveground biomass (AGBM) and belowground biomass (BGBM) in the three study species. (a) Total N (%) in lowbush blueberry (LB) in mixtures with poverty oat grass (POG). (b) Total N (%) in LB in mixtures with sweet fern (SF). (c) Total N (%) in POG in mixtures with LB. (d) Total N (%) in SF in mixtures with LB. Values are mean ± SD (*n* = 3). Values not sharing the same letters are significantly different at *P* < 0.05.

Mean N concentration in aboveground vegetative biomass across all weed density levels was lower in poverty oat grass (0.78 ± 0.12%) than in lowbush blueberry (1.15 ± 0.04%) and sweet fern (1.22 ± 0.06%). Weed density had no effect on lowbush blueberry aboveground biomass (stem + leaves) N concentrations (Table 3) but N concentrations were significantly lower in plots with poverty oat grass than in those with sweet fern (Fig 2A) (1.02 ± 0.13% vs. 1.28 ± 0.23%).

**Table 3:** Results of the mixed model analysis conducted on N concentration (%) in lowbush blueberry (LB) and weed aboveground (AGBM N_conc_) and belowground biomass (BGBM N_conc_).

Response variable	Explanatory variable	Df	Sum of square	F-value	P-value
LB AGBM N_conc_	Weed species	1	0.43	17.40	**9 10**^**−4**^
	D level	3	0.03	0.41	0.75
	D level ×Weed species	3	0.052	0.70	0.57
LB BGBM N_conc_	Weed species	1	0.042	6.68	**0.02**
	D level	3	0.026	1.38	0.29
	D level × Weed species	3	0.031	1.65	0.22
Weed AGBM N_conc_	Weed species	1	0.53	10.0	**0.01**
	D level	3	0.16	1.0	0.44
	D level × Weed species	3	0.10	0.6	0.62
Weed BGBM N_conc_	Weed species	1	2.7	2.7	**0.02**
	D level	3	0.8	0.3	0.57
	D level × Weed species	3	0.6	0.3	0.48

Weed species and weed density level were used as fixed effects and blocks as the random effect. The table shows the results of type III analyses of variance computed via Satterthwaite's degree of freedom method. Significant effects (P<0.05) are shown in bold characters.

Mean belowground biomass N concentration was higher in sweet fern (1.36 ± 0.45%) than in poverty oat grass (0.59 ± 0.02%) and lowbush blueberry (0.30 ± 0.04%). Nitrogen concentration in lowbush blueberry belowground biomass was significantly lower in association with poverty oat grass (0.26 ± 0.03) than in association with sweet fern (0.34 ± 0.12) (Table 3; Fig 2B).

Nitrogen concentration was higher in sweet fern than in poverty oat grass both in aboveground and belowground biomass (Fig 2C and 2D). Weed density had no effect on weed tissues N concentration (Table 3).

### Percentage of fertilizer-derived N recovered (PFNR) in blueberry and weeds

**Fig 3:**
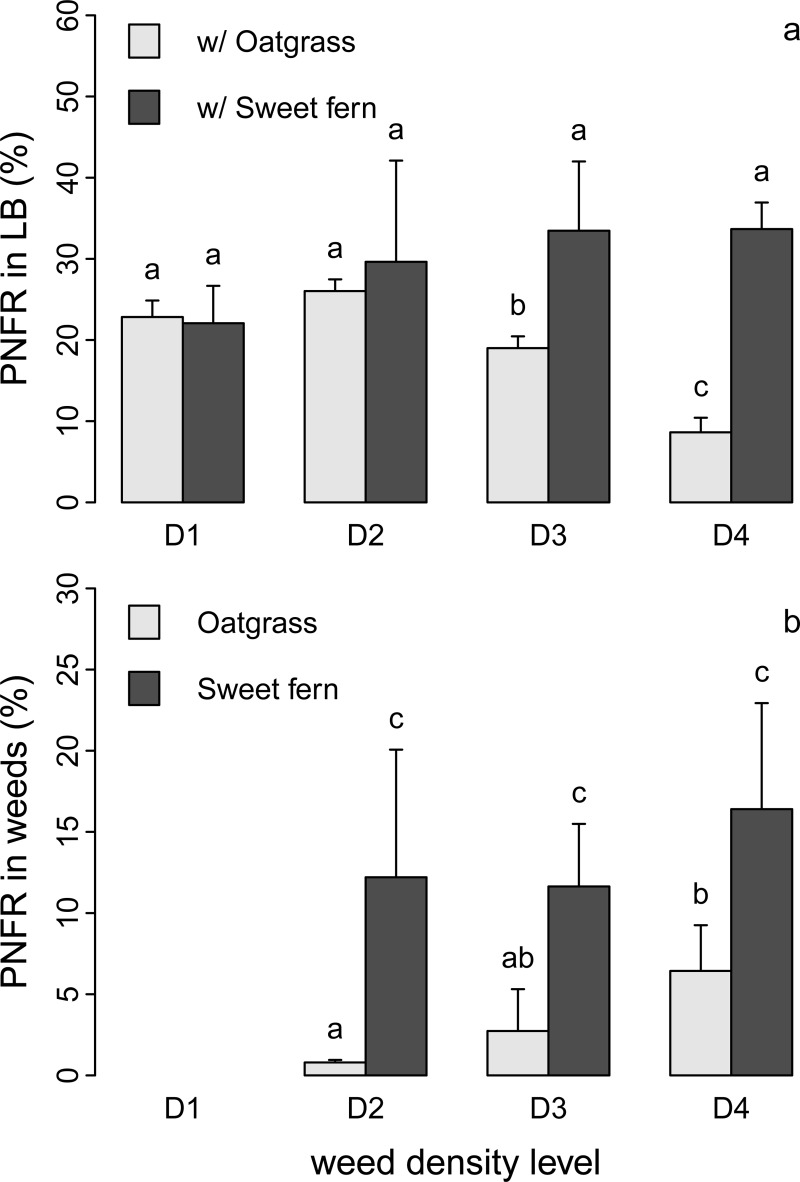
Proportion of fertilizer-derived N recovered (PFNR) in the three study species. (a) PFNR in lowbush blueberry (LB) in mixtures with poverty oat grass (POG) or sweet fern (SF). (b) PFNR in POG and SF in mixtures with LB. Values are mean ± SD (*n* = 3) and are shown for the four levels of weed density (D1-D4). Values not sharing the same letters are significantly different at *P* < 0.05.

PFNR was much higher in lowbush blueberry (24.4 ± 9.3%) than in sweet fern (13.4 ± 5.9%) and poverty oat grass (3.3 ± 3.1%). The PFNR in lowbush blueberry was similar in association with either poverty oat grass or sweet fern at low weed density levels (D1 and D2), ranging from 22.1% to 29.6% (Fig 3A). However, whereas the PFNR in lowbush blueberry did not decrease with increasing sweet fern density, it decreased significantly with increasing density of poverty oat grass (Fig 3A).

The PFNR in sweet fern did not increase significantly with plant density, taking up between 11.6% and 16.4% of fertilizer-derived N across all density levels (Fig 3B). In contrast, the PFNR in poverty oat grass did increase with density.

### Fertilizer uptake efficiency in blueberry and weed species

**Fig 4:**
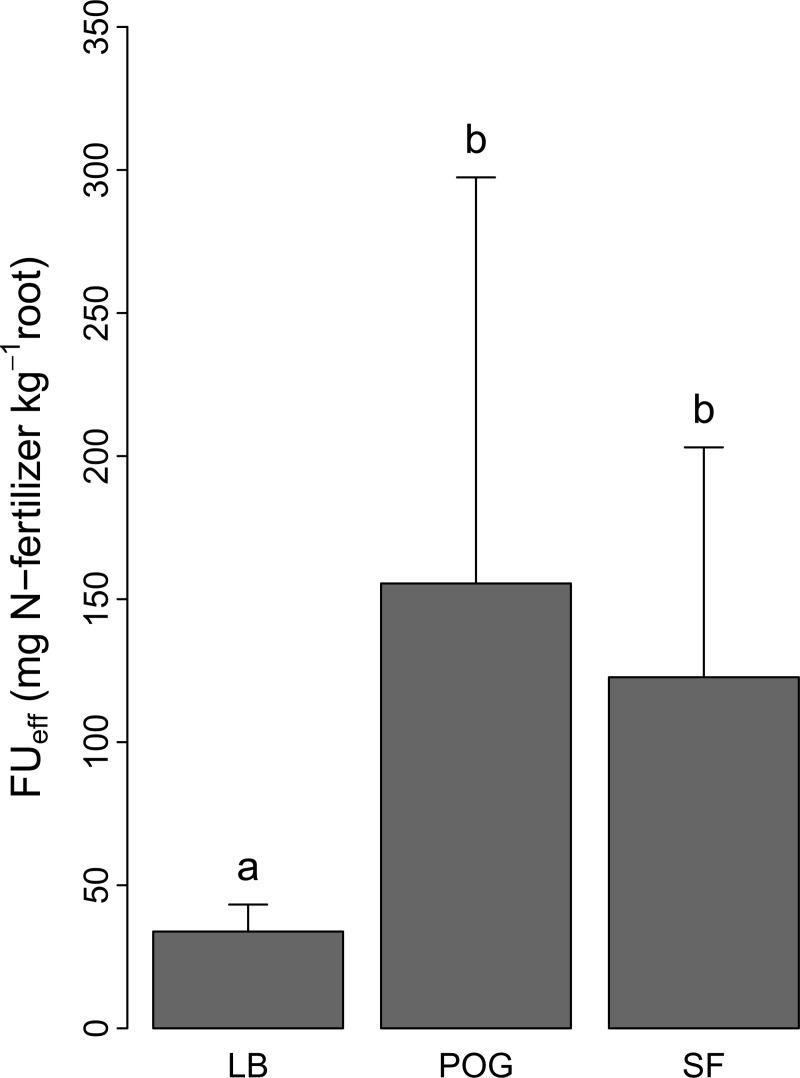
Fertilizer uptake efficiency (*FU*_*eff*_) (mg N kg^-1^ root) of lowbush blueberry (LB), sweet fern (SF) and poverty oat grass (POG). Values are mean ± SD across weed density levels. Values not sharing the same letters are significantly different at *P* < 0.05.

Fertilizer uptake efficiency *FU*_*eff*_, i.e. uptake of fertilizer-derived N per unit of root mass, was about four times higher in both sweet fern (129.8 ± 81.8 mg N-fertilizer kg^-1^ root) and poverty oat grass (155.5 ± 149.9 mg N-fertilizer kg^-1^ root) than in lowbush blueberry (33.3 ± 9.4 mg N-fertilizer kg^-1^ root) (Fig 4).

### Relationship between PFNR and plant biomass

**Fig 5:**
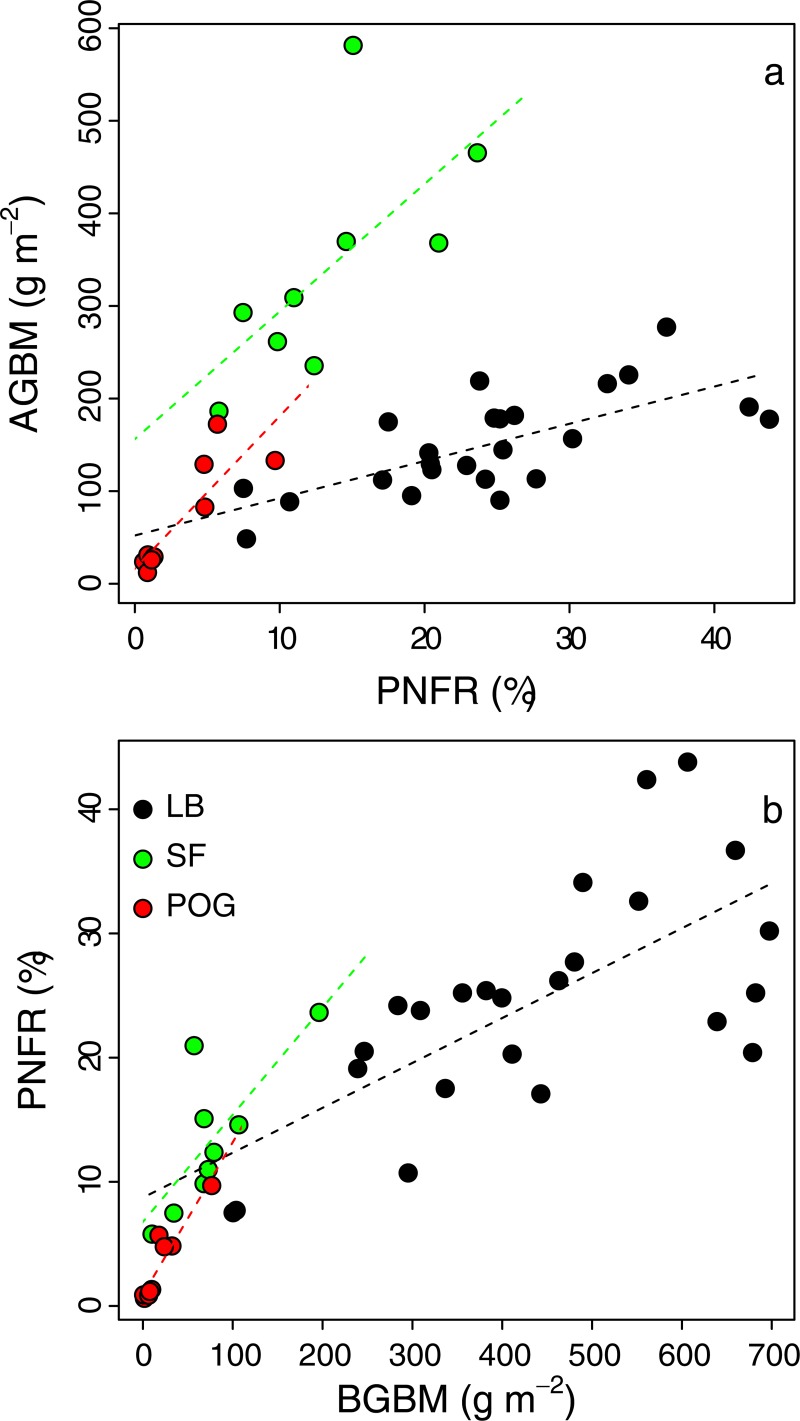
Relationships between the percentage of fertilizer-derived N recovered (PFNR) and aboveground biomass (AGBM; g m^-2^) and belowground biomass (BGBM; g m^-2^) in the three study plant species. (a) Relationship between PFNR and AGBM; y = 52.12 + 4.02x (*R*^2^ = 0.49; *P* < 0.001) for LB; y = 156.2 + 13.77x (*R*^2^ = 0.44; *P* = 0.05) for SF; y = 16.38 + 16.46x (*R*^2^ = 0.74; *P* = 0.005) for POG. (b) Relationship between BGBM and PFNR; y = 8.72 + 0.036x (*R*^2^ = 0.47; *P* < 0.001) for LB; y = 6.77 + 0.086x (*R*^2^ = 0.58; *P* < 0.05) for SF; y = 0.91 + 0.123x (*R*^2^ = 0.87; *P* < 0.001) for POG. LB, SF and POG stand for lowbush blueberry, sweet fern and poverty oat grass, respectively.

There was a positive correlation between aboveground biomass production and PFNR for both lowbush blueberry and the weed species (Fig 5A); however, the slope of this relationship was significantly higher for the weed species (13.8 and 16.5 g m^-2^ PFNR^-1^), for sweet fern and poverty oat grass, respectively) than for lowbush blueberry (4.0 g m^-2^ PFNR^-1^). The intercept was higher for sweet fern (156.2 g m^-2^) than for poverty oat grass (16.4 g m^-2^) and lowbush blueberry (52.1 g m^-2^) (S1 Table). There was also a strong positive relationship between belowground biomass and PFNR for the three species (Fig 5B). There was a significant species effect but no significant interaction between belowground biomass and PFNR (S2 Table), although the slopes tended to be higher for the weed species (0.086 and 0.123 PFNR g m^-2^ in sweet fern and poverty oat grass, respectively) than for lowbush blueberry (0.036 PFNR g m^-2^).

## Discussion

### Fertilizer uptake by lowbush blueberry and weed species

Fifteen months after the application of ^15^N-labelled fertilizer, a much larger proportion of ^15^N was recovered in lowbush blueberry than in both weed species. The strong and positive relationship between belowground biomass and PFNR for all species (Fig 5B) shows that this higher fertilizer acquisition resulted from a much higher belowground biomass in lowbush blueberry than in the weed species. Lowbush blueberry also produced more aboveground biomass than both weed species, but this production was low relative to belowground biomass. More than 10 g of belowground biomass was required to produce 1 g of aboveground biomass as compared with only 0.5 and 0.7 g for poverty oat grass and sweet fern, respectively. The lower slope coefficient of the linear regression between PFNR and aboveground biomass production in lowbush blueberry than for the weed species reflected a lower fertilizer-derived N use efficiency for lowbush blueberry (Fig 5A). A greater amount of fertilizer was necessary to produce a given amount of aboveground biomass in lowbush blueberry than in the weed species. The intercept of the regression line represents the theoretical aboveground biomass production in the absence of fertilizer-derived N. This parameter was much higher in sweet fern than in poverty oat grass and lowbush blueberry, suggesting that sweet fern has access to other sources of N to support its high aboveground biomass production. A major portion of this N likely came from atmospheric N-fixation since sweet fern has a symbiotic association with *Frankia*, a N-fixing actinobacterium; in addition, sweet fern’s deep and clustered root system may give this weed access to soil ammonium and nitrate present in the soil, albeit at very low concentration [32,33]. In contrast, the very low intercept (close to zero) for poverty oat grass suggests that this weed is much more dependent on the N fertilizer to support its growth. These results agree with our hypotheses that i) the dense and superficial root system of poverty oat grass allows it to take up the fertilizer efficiently; and ii) sweet fern does not rely upon the fertilizer to support its N demand as much as poverty oat grass due to its ability to fix atmospheric N.

Although lowbush blueberry captured most of the added inorganic N, our results show that lowbush blueberry’s root system is inefficient in acquiring fertilizer-derived inorganic N relative to that of both weed species, especially poverty oat grass. Fertilizer uptake efficiency, i.e. fertilizer uptake normalized with belowground biomass, was about four times lower in lowbush blueberry than in both weed species (Fig 4). Thus, lowbush blueberry must invest four times more resources and energy in its belowground biomass than the weed species to acquire equivalent amounts of inorganic N from fertilizer. This low nutrient absorption efficiency is common to most blueberry species and results from a low proportion of root hairs [42]. In addition, ericaceous species have low N requirements as they have evolved adaptations to survive in habitats having low inorganic N availability and mineralization rates, especially cold climate species, such as lowbush blueberry [43]. Their use of nitrate is generally insignificant because ericaceous species have small or null nitrate reductase activity in their leaves and roots [44,45]. Ericaceous species rather support their N demand by taking up organic N molecules through their ericoid mycorrhizal associations [9,44,46]. Although several studies have shown that ammonium inputs increase lowbush blueberry growth and N status [7,11,12,14], our results support the hypothesis of a lower N fertilizer uptake and use efficiency in lowbush blueberry than in the two weed species. This low fertilization uptake ability of blueberry species generally results in low fertilizer recovery in ^15^N-labelling experiments (~5–40% depending on crop management, species and on the phenological stage the tracer is applied) [47,48]. A recent study has shown that unadapted N fertilization (fertilization rate, timing and type) could lead to decreased yields, increased soil electrical conductivity and nitrate leaching in highbush blueberry fields [49]. Our results show that N fertilizer should also be used with caution, depending on local neighboring species and densities. Adding N fertilizer may be counterproductive in situations where the lowbush blueberry root system is not well developed and where poverty oat grass is present at a high density. Our data show that adding N fertilizer in areas where poverty oat grass density >10 plants m^-2^ may indeed provide a greater benefit to poverty oat grass and have a detrimental effect on lowbush blueberry growth. In contrast, N fertilization may not require systematic weed suppression in areas where poverty oat grass density is low (<10 plants m^-2^) and where sweet fern is the sole weed species, as these conditions did not impact lowbush blueberry’s fertilizer uptake.

### Interactions between lowbush blueberry and the weed species

In agreement with our hypotheses, sweet fern and poverty oat grass interacted differently with lowbush blueberry. First, lowbush blueberry vegetative biomass was higher in the plots with sweet fern than in those with poverty oat grass (Fig 1). Second, whereas both aboveground and belowground biomass of lowbush blueberry tended to decrease with increasing poverty oat grass density, lowbush blueberry aboveground vegetative biomass production tended to increase with sweet fern density; this resulted in the significant interaction between weed species and weed density (Table 2). A part of these opposite trends could be explained by the higher lowbush blueberry belowground biomass in association with sweet fern than with poverty oat grass (there was a positive relationship between belowground biomass and aboveground vegetative biomass production in lowbush blueberry: *r* = 0.55; *P* < 0.005), rather than from a stimulation of lowbush blueberry aboveground vegetative biomass by sweet fern. Indeed, the production of aboveground vegetative biomass per unit root biomass was similar across density levels and in both mixture types (0.11 ± 0.06 and 0.09 ± 0.02 in poverty oat grass and sweet fern, respectively). This absence of change in the aboveground: belowground biomass ratio across the different treatments (weed species and weed density) contrasts with other studies reporting changes in biomass allocation in response to competition with other species and resources limitations [50,51].

It is not clear why lowbush blueberry belowground biomass was higher in plots with sweet fern than in those with poverty oat grass. However, the fact that this trend was true even in plots with very few or no weeds (i.e. 0–1 plant m^-2^ in D1 plots) suggests that sweet fern may have an indirect effect on lowbush blueberry root growth, by for instance changing soil physico-chemical characteristics in the long-term (e.g., soil N concentration, soil structure). Yet, available soil data seem to indicate that soil is more nutrient-depleted in areas colonized by sweet fern than in those colonized by poverty oat grass (S1 Table). More research is required to clarify this point. It is also not clear why this biomass decreased with increased poverty oat grass density, whereas no such trend was observed with sweet fern (Fig 1A and 1B). This pattern may be a result of lower competition for root development in association with sweet fern than in association with poverty oat grass, or it may simply be due to sweet fern colonizing the field later when lowbush blueberry belowground biomass was already well developed. However, sweet fern tends to colonize lowbush blueberry fields earlier than poverty oat grass (personal observation). As such, we believe that lowbush blueberry root development is hampered more by poverty oat grass than by sweet fern. In addition, the decrease in PFNR in lowbush blueberry with increasing poverty oat grass density is evidence of competition between these two species for acquiring fertilizer-derived N. This competition was probably responsible for the decrease in lowbush blueberry aboveground vegetative biomass production with increasing poverty oat grass density as suggested by the strong correlation between aboveground vegetative biomass and PFNR (Fig 5A). In contrast, the lack of impact of sweet fern density on PFNR in lowbush blueberry suggests that sweet fern scarcely compete for acquiring N. In addition, the higher N concentrations in lowbush blueberry tissues in sweet fern plots suggest that the latter species even facilitates N acquisition by lowbush blueberry and possibly its biomass production.

Unsurprisingly, biomass N content in both weed species tended to increase with increasing plant density (S1 Fig). However, while this increase was accompanied by an increase in PFNR in poverty oat grass, this was not the case for sweet fern (i.e., constant PNFR); thus, sweet fern used increasingly another source of N to support its growth as its density increased, most likely an increased contribution of atmospherically-derived N. Several studies have shown that N_2_-fixing species tend to derive a higher proportion of their N from the atmosphere as competition for acquiring soil N increased [26,52–54]. Conversely, this proportion often decreases with increased N fertilization [27,54]. Therefore, increased competition for soil N due to increased plant density (lowbush blueberry + sweet fern) and the resulting increased demand for N can explain sweet fern’s increased reliance upon N_2_-fixation. This mechanism can produce “N sparing”, i.e. increased inorganic N availability for neighboring species [28,29] and explain why neither lowbush blueberry aboveground biomass production nor PFNR decreased with increased sweet fern density. This use of atmospheric N_2_ as a N source can explain the higher intercept of the regression line of the relationship between aboveground biomass production and PFNR (Fig 5A) and reveals that sweet fern can produce a larger amount of biomass with less contribution from fertilizer-derived N. The interaction between lowbush blueberry and sweet fern resulted in an improved use of N as shown by higher plant biomass production per surface unit and a higher proportion of ^15^N recovered in lowbush blueberry + sweet fern plots (43%) than in lowbush blueberry + poverty oat grass plots (22%).

## Conclusion

Our data show that lowbush blueberry is not as efficient as weed species at acquiring inorganic N from the fertilizer. In our experiment, lowbush blueberry captured most of the added fertilizer only because individual plants had a well-developed root system. Fertilization in young lowbush blueberry stands having an undeveloped root system may not stimulate lowbush blueberry growth, especially when poverty oat grass density is >10 plants m^-2^, in which case fertilization may benefit the weed species more than the targeted plant. Our results show that poverty oat grass relies strongly on the fertilizer to fulfill its N demand relative to the other studied species. At plant densities >25 plants m^-2^, poverty oat grass captured about the same amount of fertilizer as blueberry despite a much smaller belowground biomass, resulting in a significant negative impact on blueberry’s vegetative biomass. In contrast, sweet fern uses atmospherically-derived N to support its N demand, especially when present at high density. This likely resulted in a “N sparing” phenomenon that alleviated the competition for acquiring N and explained the absence of detrimental effect of sweet fern density on lowbush blueberry fertilizer-derived N uptake. Based on our results, we recommend poverty oat grass suppression before applying N fertilizer in areas where poverty oat grass density is >10 plants m^-2^. In contrast, fewer precautions are required for applying fertilizer in plots having sweet fern, as this species does not compete with lowbush blueberry for acquiring N. Further research is required to assess the impacts of both weeds on lowbush blueberry fruit production.

## Supporting information

S1 Fig Aboveground vegetative biomass (AGVBM) and belowground biomass (BGBM) N content (g N m^-2^) in the three study plant species. (a–d) N content in lowbush blueberry (LB) AGVBM and BGBM in mixtures with poverty oat grass (POG; dark grey bars) or sweet fern (SF; light grey bars). (e–h) N content in POG and SF AGVBM and BGBM in mixtures with LB. Values are mean ± SD (*n* = 3) and are shown for the four levels of weed density (D1-D4). Values not sharing the same letters are significantly different at *P* < 0.05.
(DOCX)

S1 Table Soil characteristics in the study lowbush blueberry field. Data are shown for the two areas of the field where the plots were located: the area colonized by sweet fern and the area colonized by poverty oat grass.
(DOCX)

S2 Table Results of analysis of covariance (ANCOVA) conducted on plants’ aboveground vegetative biomass (AGVBM) production. Plant species was used as a categorical variable and the percentage of fertilizer-derived N recovered (PFNR) in plants as a covariate.
(DOCX)

S3 Table Results of an analysis of covariance (ANCOVA) conducted on the percentage of fertilizer-derived N recovered (PFNR) in plants. Plant species was used as a categorical variable and the belowground biomass (BGBM) as a covariate.
(DOCX)

S1 Data Raw data for N concentration (%) and ^15^N/^14^N ratio in the aboveground and belowground biomass (g m^-2^) of lowbush blueberry, sweet fern and poverty oat grass as a function of weed density level and companion species. (XLSX)

S2 Data Aboveground and belowground biomass (g m^-2^), N concentration (%) and percentage of fertilizer-derived N recovered [PFNR (%)] in lowbush blueberry, sweet fern and poverty oat grass as a function of weed density level and companion species. (CSV)
