# Supplementary material for: Contrasting impacts of two weed species on lowbush blueberry fertilizer nitrogen uptake in a commercial field
Source: PLoS One. 2019 Apr 12;14(4):e0215253. doi: 10.1371/journal.pone.0215253 (PMC6461287; doi:10.1371/journal.pone.0215253)
Supplement: S1 Fig — (a–d) N content in lowbush blueberry (LB) AGVBM and BGBM in mixtures with poverty oat grass (POG; dark grey bars) or sweet fern (SF; light grey bars). (e–h) N content in POG and SF AGVBM and BGBM in mixtures with LB. Values are mean ± SD (n = 3) and are shown for the four levels of weed density (D1-D4). Values not sharing the same letters are significantly different at P < 0.05. (DOCX) [file pone.0215253.s001.docx]

S1 Figure. Aboveground vegetative biomass (AGVBM) and belowground biomass (BGBM) N content (g N m^-2^) in lowbush blueberry (LB) in mixture with poverty oat grass (POG) or sweet fern (SF) (a–d); and in POG or SF in mixture with LB (e–h) at four levels of weed density (D1–D4). Values are mean ± SD (*n* = 3). Values not sharing the same letters are significantly different at *P* < 0.05.
