# Supplementary material for: Contrasting impacts of two weed species on lowbush blueberry fertilizer nitrogen uptake in a commercial field
Source: PLoS One. 2019 Apr 12;14(4):e0215253. doi: 10.1371/journal.pone.0215253 (PMC6461287; doi:10.1371/journal.pone.0215253)
Supplement: S1 Table — Data are shown for the two areas of the field where the plots were located: the area colonized by sweet fern and the area colonized by poverty oat grass. (DOCX) [file pone.0215253.s002.docx]

S1 Table. Soil characteristics in the study lowbush blueberry field. Data are shown for the two areas of the field where the plots were located: the area colonized by sweet fern and the area colonized by poverty oat grass.

|  | #Sweet fern | #Poverty oat grass |
| --- | --- | --- |
| CEC (meq/100g) | 16.5 | 17.6 |
| pH_water (1:1)_ | 5.1 | 4.7 |
| Ca (Mehlich III) (ppm) | 131 | 169 |
| Saturation Ca (%) | 4.0 | 5.0 |
| P (Mehlich III) (ppm) | 27 | 67 |
| Al (Mehlich III) (ppm) | 895 | 799 |
| K (Mehlich III) (ppm) | 23 | 35 |
| Saturation K (%) | 0.4 | 0.5 |
| Mg (Mehlich III) (ppm) | 9 | 17 |
| Saturation Mg (%) | 0.50 | 0.86 |
| SOM (combustion) (%) | 4.2 | 4.4 |
| Base Saturation [K+Mg+Ca] (%) | 4.8 | 6.2 |
