# Supplementary material for: Contrasting impacts of two weed species on lowbush blueberry fertilizer nitrogen uptake in a commercial field
Source: PLoS One. 2019 Apr 12;14(4):e0215253. doi: 10.1371/journal.pone.0215253 (PMC6461287; doi:10.1371/journal.pone.0215253)
Supplement: S2 Table — Plant species was used as a categorical variable and the percentage of fertilizer-derived N recovered (PFNR) in plants as a covariate. (DOCX) [file pone.0215253.s003.docx]

S2 Table. Results of analysis of covariance (ANCOVA) conducted on plants’ aboveground vegetative biomass (AGVBM) production with species as a categorical variable and the percentage of fertilizer-derived N recovered (PFNR) in plants as a covariate.

|  | Df | Sum of squares | *F*-value | *P*-value |
| --- | --- | --- | --- | --- |
| PFNR | 1 | 48207 | 16.1549 | 0.0002849*** |
| Species | 2 | 385446 | 64.5842 | 1.232e-12*** |
| PFNR × Species | 2 | 32888 | 5.5106 | 0.0081687** |
| Residuals | 36 | 107426 |  |  |
