# Supplementary material for: Contrasting impacts of two weed species on lowbush blueberry fertilizer nitrogen uptake in a commercial field
Source: PLoS One. 2019 Apr 12;14(4):e0215253. doi: 10.1371/journal.pone.0215253 (PMC6461287; doi:10.1371/journal.pone.0215253)
Supplement: S3 Table — Plant species was used as a categorical variable and the belowground biomass (BGBM) as a covariate. (DOCX) [file pone.0215253.s004.docx]

S3 Table. Results of an analysis of covariance (ANCOVA) conducted on the percentage of fertilizer-derived N recovered (PFNR) in plants with species as a categorical variable and the belowground biomass (BGBM) as a covariate.

|  | Df | Sum of squares | *F*-value | *P*-value |
| --- | --- | --- | --- | --- |
| PFNR | 1 | 3917.9 | 119.5545 | 5.421e-13*** |
| Species | 2 | 276.2 | 4.2138 | 0.02268* |
| PFNR × Species | 2 | 86.0 | 1.3128 | 0.28163 |
| Residuals | 36 | 1179.8 |  |  |
